# Protocol for combined N-of-1 trials to assess cerebellar neurostimulation for movement disorders in children and young adults with dyskinetic cerebral palsy

**DOI:** 10.1186/s12883-024-03633-z

**Published:** 2024-04-29

**Authors:** M San Luciano, C R Oehrn, S S Wang, J S Tolmie, A Wiltshire, R E Graff, J Zhu, P A Starr

**Affiliations:** 1 Movement Disorders and Neuromodulation Center, Department of Neurology, University of California San Francisco, Weill Institute for Neurosciences, 1651 4th Street Level 3 SW Academic Offices, Box #1838, 94158 San Francisco, CA USA; 2 Department of Neurological Surgery, University of California San Francisco, San Francisco, CA USA; 3 Department of Epidemiology and Biostatistics, University of California San Francisco, San Francisco, CA USA

**Keywords:** Dyskinetic cerebral palsy, Children, Young adults, Cerebellum, Dentate nucleus, Deep brain stimulation, Electrophysiology

## Abstract

**Background:**

Movement and tone disorders in children and young adults with cerebral palsy are a great source of disability. Deep brain stimulation (DBS) of basal ganglia targets has a major role in the treatment of isolated dystonias, but its efficacy in dyskinetic cerebral palsy (DCP) is lower, due to structural basal ganglia and thalamic damage and lack of improvement of comorbid choreoathetosis and spasticity. The cerebellum is an attractive target for DBS in DCP since it is frequently spared from hypoxic ischemic damage, it has a significant role in dystonia network models, and small studies have shown promise of dentate stimulation in improving CP-related movement and tone disorders.

**Methods:**

Ten children and young adults with DCP and disabling movement disorders with or without spasticity will undergo bilateral DBS in the dorsal dentate nucleus, with the most distal contact ending in the superior cerebellar peduncle. We will implant Medtronic Percept, a bidirectional neurostimulator that can sense and store brain activity and deliver DBS therapy. The efficacy of cerebellar DBS in improving quality of life and motor outcomes will be tested by a series of N-of-1 clinical trials. Each N-of-1 trial will consist of three blocks, each consisting of one month of effective stimulation and one month of sham stimulation in a random order with weekly motor and quality of life scales as primary and secondary outcomes. In addition, we will characterize abnormal patterns of cerebellar oscillatory activity measured by local field potentials from the intracranial electrodes related to clinical assessments and wearable monitors. Pre- and 12-month postoperative volumetric structural and functional MRI and diffusion tensor imaging will be used to identify candidate imaging markers of baseline disease severity and response to DBS.

**Discussion:**

Our goal is to test a cerebellar neuromodulation therapy that produces meaningful changes in function and well-being for people with CP, obtain a mechanistic understanding of the underlying brain network disorder, and identify physiological and imaging-based predictors of outcomes useful in planning further studies.

**Trial registration:**

ClinicalTrials.gov NCT06122675, first registered November 7, 2023.

## Background

Cerebral palsy (CP), an umbrella term for a group of disorders and posture caused by non-progressive brain disturbances [1], represents the most common physical disability of childhood, affecting one out of 323 children in the United States [1, 2]. Movement disorders, including dystonia and choreoathetosis, occur in 15–17% of CP cases, a condition known as dyskinetic CP (DCP) [3]. Mixed presentations are also common, as dystonia may be present to some degree in other CP forms, and DCP frequently has co-morbid spasticity [4].

The treatment of movement disorders in DCP is challenging and frequently fails to meet patient needs. Oral medications generally have low efficacy in DCP and are limited by side effects [5, 6]. Intrathecal baclofen improves dystonia, spasticity, and pain. However, it carries a high risk of infection and surgical complications compared to other surgical options, including deep brain stimulation (DBS) surgery [7]. Selective dorsal rhizotomies, surgical interventions for predominantly lower limb spasticity, do not help dystonia and may even exacerbate it [8].

DBS surgery targeting the globus pallidus and motor thalamus has been increasingly used in individuals with acquired dystonia, including DCP, when other treatments are not effective or tolerated [9, 10]. DBS is an invasive neuromodulation technique that targets pathological brain circuitries [11], and entails the implantation of bilateral electrodes into deep brain targets for the delivery of an electrical current via an impulse generator implanted in the chest. Pallidal and subthalamic DBS is Food and Drug Administration (FDA) approved under a Humanitarian Device Exemption in the United States for the treatment of chronic treatment-refractory primary dystonia. DBS offers advantages over other surgical therapies, including safety, reversibility, tolerability, and adaptability to the individual patient. DBS in traditional targets (globus pallidus and motor thalamus) can improve motor, disability and pain ratings in DCP, but motor and quality of life outcomes are highly variable and generally less beneficial than in isolated dystonia [5, 6, 9, 12–14]. The relatively reduced benefit is thought to be partially related to implantation in targets with pre-existing structural damage and lack of effect on comorbid choreoathetosis and spasticity [6].

The cerebellum represents an attractive target for DBS in DCP since it has an increasingly recognized role in dystonia pathophysiology [15] and is frequently spared from hypoxic ischemic damage in DCP [16]. Stimulation of cerebellar pathways improves dystonia in pre-clinical genetic animal models [17].

Cerebellar DBS for dystonia and spasticity in DCP arose from early reports of lesion-based surgeries and electrical stimulation published in the 1960s and 1970s [18–21]. The reports showed an average 60–70% long-term improvement, although concerns existed regarding stimulator reliability and safety [22]. More recent evidence using cylindrical DBS leads in the dentate nucleus have shown preliminary improvements in dystonia/choreoathetosis and even spasticity [18, 22–27]. We recently reported surgical notes and preliminary outcomes in three subjects with DCP with bilateral cerebellar DBS, targeting the motor dentate nucleus and cerebellar outflow pathways [24]. These individuals demonstrated improvements in objective movement rating scales, and subjective improvements in function without worsening in coordination. Other groups have reported successful and safe cerebellar DBS for other conditions, including cerebellar ataxia [28].

Here, we present the protocol for a single-center series of randomized, double-blinded N-of-1 trials studying cerebellar DBS for the treatment of severe movement and tone disorders in ten children and young adults with DCP. This trial is currently open for recruitment at the University of California, San Francisco (UCSF).

## Methods/Design

### External review of study protocol

The study protocol has been reviewed and approved by both the National Institutes of Health’s National Institute of Neurological Disorders and Stroke (grant number UH3NS128297, notice of award 03/13/2024) and the Food and Drug Administration (investigational device exemption no. G230261; December 15, 2023).

### Study objectives

This study aims to ameliorate severe movement and tone disorders in CP by chronic neuromodulation (DBS) of motor dentate nuclei and cerebellar output pathways. The efficacy of cerebellar stimulation for improving quality of life (QOL) and motor outcomes will be tested by periodic clinical assessments and objective kinematic metrics. In addition, we will characterize abnormal patterns of cerebellar oscillatory activity as measured by local field potentials (LFP), and evaluate candidate imaging markers of baseline disease severity and response to DBS by performing pre- and 12-month postoperative volumetric structural and functional MRIs.

### Study design and setting

This is a single-center series of randomized, double-blinded N-of-1 trials to test the effects of chronic DBS of dentate and cerebellar output pathways on severe movement and tone disorders in DCP. This study will recruit ten children and young adults aged 7–25 with DCP secondary to hypoxic ischemic static encephalopathy (HIE). We will implant Medtronic Percept™, an FDA-approved “bidirectional” neurostimulator that can sense and store brain activity while delivering DBS therapy.

**Fig. 1:**
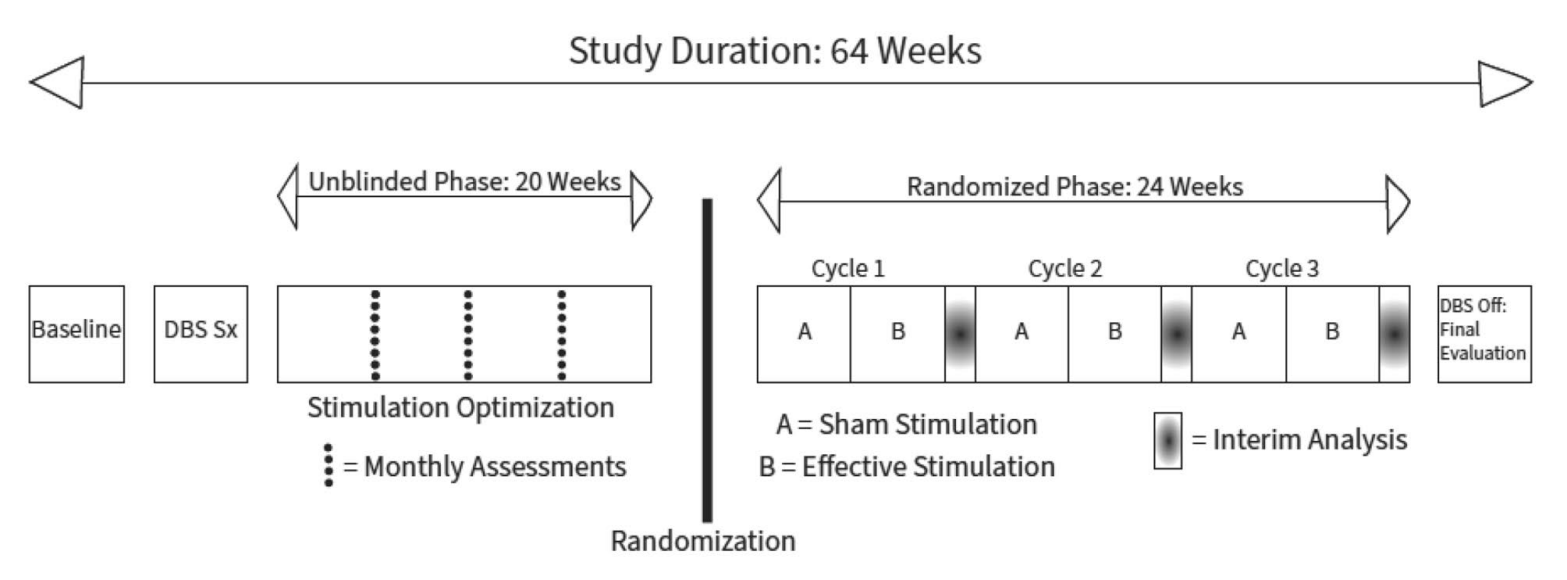
Study outline

We will use a series of N-of-1 clinical trials to enhance causal inference and mitigate the variability in clinical features in this population (Fig. 1). We will use a cross-over design and compare three months of effective stimulation with three months of sham stimulation for each patient. We will administer the treatments in blocks of two months with one month per condition (sham vs. effective stimulation) in a randomized order. During these one-month trials for each condition, patients, caregivers, and assessing clinicians will be blinded to the state of the stimulator, and outcomes will be measured at weekly intervals. To evaluate kinematic metrics, we will use videotaped automated movement recognition techniques and formal gait analysis.

This protocol was developed to adhere to the Standard Protocol Items: Recommendations for Interventional Trials (SPIRIT) extension for N-of-1 trials (SPENT) checklist that is aligned with the CONSORT (consolidated reporting items for trials) extension for N-of-1 trials (CENT, 29).

### Recruitment and participant selection

Participants will be recruited from the UCSF Movement Disorders and Neuromodulation Center and the Pediatric Neurology Clinics at UCSF Benioff Children’s Hospitals in San Francisco and Oakland, California, USA. Our UCSF Movement Disorders and Neuromodulation Center includes a multidisciplinary clinic with a special focus on surgical therapies and has a long history of successful recruitment for surgical trials in movement disorders.

The inclusion criteria include a diagnosis of DCP with or without comorbid spasticity (Gross Motor Function Classification System, GMFCS, Levels II-V), with a clear history of hypoxic ischemic brain injury preceding motor symptoms made by a pediatric neurologist, with supporting MRI findings, sparing the cerebellum. Potential participants will have a history of appropriate therapy with oral medications with inadequate relief, and severe enough movement disorders to warrant consideration for DBS therapy. Exclusion criteria include medical conditions significantly increasing surgical risks, uncontrolled epilepsy, pregnancy, severe fixed contractions and skeletal deformities, and CP etiologies apart from HIE (for example, genetic mimics of cerebral palsy, absence of documented risk factors for HIE, traumatic brain injury or history of infectious or autoimmune encephalitis). As of 04/05/2024, we have successfully recruited one patient, who is scheduled for surgery in the upcoming months.

### Informed consent

All participants are required to give informed consent prior to enrollment. Assent will be sought from all involved minor participants and adults who are not capable of consent. The context of the specific neurological condition and neurosurgical treatment proposed in this research project may make it difficult for patients and families to distinguish between procedures that are essential to their clinical care and similar procedures that are undertaken solely for research purposes. In this study, participants may have impaired cognition and need careful assessment of capacity for research participation as well as for treatment. For this purpose, this study will use a “teach-to-goal” consent framework [30–32]. Teach-to-goal is an educational strategy in which the informed consent document is reviewed with prospective participants, who then answer questions about critical elements of the study. Misperceptions are corrected and the participant’s comprehension is assessed again. Consent from a surrogate from those individuals who cannot demonstrate comprehension after several attempts is then sought. Adult patients, especially those without known intellectual disability, will be presumed to have capacity to consent. However, if an adult patient is known to have intellectual disability or if during the consent process, questions arise regarding the ability to consent, the capacity to consent to participate in the study will be formally determined.

### Retention plan

Our retention strategies include: maximizing convenience for patients by integrating research visits with routine clinical care wherever possible, having all study clinicians, researchers, and coordinators in a single location, ensuring cost-neutrality for the participant and family, and maintaining motivation by providing frequent updates to participants and their families on the progress of the study.

### Study outcomes

For this clinical trial, there is a primary QOL and a primary motor outcome. The QOL endpoint will be the within- and between-child difference in Caregiver Priorities & Child Health Index of Life with Disabilities (CPCHILD) QOL scale total standardized score [33], while on intervention (effective stimulation) versus placebo (sham stimulation). The rationale for this QOL primary outcome measure is that health-related QOL measures provide a more complete picture of an individual than motor assessments alone. Health- related QOL measures have been shown to help medical decision-making and have been used in practice to evaluate patients’ responses to interventions and allow providers to focus on the areas that are most important for the child and family [13]. The primary motor outcome will be the within- and between-child difference in the global index score of the Movement Disorder Childhood Rating Scale 4–18 Revised (MD-CRS 4-18-R [34], ). This is a scale specifically designed for the assessment of movement disorders for children and young adults with secondary dyskinetic movement disorders such as DCP [34].

Key secondary outcome measures will be individual CPCHILD domain scores, other health-related QOL scores (Burke-Fahn-Marsden Dystonia Rating Disability subscale, PedsQL™ scores, and Clinician and Patient Global Impression of Change scales), other motor and spasticity ratings (Burke-Fahn-Marsden Dystonia Rating Motor subscale, Unified Dystonia Rating Scale, Dyskinesia Impairment Scale, Hypertonia Assessment Tool, and Modified Ashworth Scale scores), and exploratory automatic video kinematic and gait analyses. See Table 1 for a summary of assessments.

**Table 1:** Study assessment battery

Name	Description
**Motor Assessments**	
Movement Disorder-Childhood Rating Scale 4–18 Revised (MD-CRS 4-18-R)	The MD-CRS 4–18 R is a validated tool aimed to evaluate movement disorders in the developmental age. It is particularly useful for rating the severity of movement disorders in dyskinetic cerebral palsy.
Burke-Fahn-Marsden Dystonia Rating Scale, Motor subscale (BFMDRS-M)	The BFMDRS is a universally applied biomarker for the severity of dystonia. The scale consists of a movement and disability subscale. The movement subscale rates dystonia severity and provoking factors in nine body areas (eyes, mouth, speech and swallowing, neck, trunk, arms and legs).
Unified Dystonia Rating Scale (UDRS)	The UDRS is a validated and detailed assessment of the severity of dystonia in 14 individual body areas. Each region is rated for dystonia severity and duration, and ratings for each region are totaled for an overall severity rating.
Hypertonia Assessment Tool (HAT)	The HAT is a 7-item clinical assessment tool used to differentiate the various types of pediatric hypertonia (dystonia, spasticity, and rigidity). It has good interrater, test-retest reliability and validity. Each limb is observed and receives an individual diagnosis of hypertonia.
Dyskinesia Impairment Scale (DIS)	The DIS assesses dystonia and choreoathetosis, consisting of one DIS total score and two subscale scores (for dystonia and choreoathetosis), evaluating the presence of either or both in 12 body regions, as well as the duration, amplitude and severity of the movement.
Modified Ashworth Scale (MAS)	The MAS is the most universally accepted clinical tool used to measure the increase of muscle tone. It is validated and widely applied in clinical practice and research as a measure of spasticity.
**Quality of Life Assessments**	
Caregiver Priorities & Child Health Index of Life with Disabilities (CPCHILD)	The CPCHILD is a reliable and valid measure of caregivers’ perspectives on health status, functional limitations, and well-being of patients with severe CP, including those individuals who are non-verbal and non-ambulatory.
Burke-Fahn-Marsden Dystonia Rating Scale, Disability subscale (BFMDRS-D)	The disability subscale is composed of 7 items for activities of daily living, including speech, writing, feeding, eating, hygiene, dressing and walking.
Pediatric Quality of Life InventoryÒ (PedsQL)	The PedsQL Scale is a modular approach to measuring health-related QOL in healthy children and adolescents and those with acute and chronic health conditions. It is brief, practical, developmentally appropriate, reliable, available and valid. The 23 item PedsQL generic core scale measures several areas including: physical, emotional, social and school functioning, summarized into a total scale score, a physical health summary score and a psychosocial health summary score.
Patient/Caregiver and Clinician Global Impression of Changes (GIC)	The GIC is a self-report measure that reflects a patient’s (or assesor’s) belief about the efficacy of treatment. It is a 7-point scale depicting a patient’s (or caregiver or clinician) rating of overall improvement, commonly used in research studies.
10 cm Visual Analog Pain Scale (VAS)	The 10 cm VAS is commonly used as a validated measure of pain in the context of CP, where 0 cm represents ‘no pain’ and 10 cm for ‘worst pain imaginable’.
**Neuropsychological battery**	
Kaufman Brief Intelligence Test, 2nd edition (KBIT-2)	The KBIT-2 is a brief reliable and validated measure of verbal and non-verbal intelligence used individuals ages 4 and older.
Behavior Assessment System for Children, 3rd edition (BASC-3)	The BASC-3 is a reliable systematic measure of behavioral and emotional functioning in children and adolescents. We will include self-report and parent-rated subscales.
Diagnostic and Statistical Manual of Mental Disorders, 5th edition (DSM-5), Mood Screener	The DSM-5 Mood Screener is a systematic self- and informant-rated measure that assesses mood domains that are important across psychiatric diagnoses.
Dysdiadochokinetic syllables	This test measures the patient’s ability to perform repetition of syllables at maximum rate of production. It is considered a routine assessment of motor components of speech difficulties.

### Sample size

The power calculation [35] is based on the more variable CPCHILD score. Standardized CPCHILD scores, which range from 0 (best) to 100 (worst), will be calculated for each of the scale’s seven domains and the total survey (calculated by dividing raw scores by the maximum item score, multiplied by 100). We chose a 10-point difference as a clinically relevant difference between sham and effective stimulation [13]. Assuming normally distributed differences and treatment-by-patient interaction standard deviation of 12.6 based on previously published data [36], relatively stable disease and minimal carry-over of effects, and a type I error rate of 0.05 (two-sided), a chosen sample size of *n* = 10 would have over 80% power to detect clinically relevant differences.

### Surgical procedure and study assessments

#### Phase 1: screening and eligibility

Potential participants will be identified from sequential patients evaluated at our UCSF Movement Disorders and Neuromodulation Center. After their initial clinic visit and surgical candidacy screening (including careful review of birth and developmental history, prior brain imaging, and other relevant work up), potential study candidates will be identified at a bimonthly group conference devoted to surgical research protocols. A study clinician will then contact eligible subjects and their families to discuss the study and provide the consent form. After an interval of at least 2 weeks to review the consent, potential study subjects and their families will have an in-person visit with study investigators to review the protocol in detail and sign the informed consent (and assent if applicable). All consented study subjects will undergo extensive characterization at their baseline visit including brain MRI, detailed general and neurological examination, standardized videotaped motor exams and QOL rating scales, and formal neuropsychological evaluations.

#### Phase 2: surgery and open-label titration phase

Study subjects will undergo DBS surgery to be implanted with a totally internalized bidirectional neural interface, Medtronic Percept and Medtronic SenSight electrodes. The stimulation is targeted to the motor dentate nucleus and the cerebellar outflow pathways with the tip of the lead at the proximal (onset) superior cerebellar peduncle, with all contacts except for the most distal one traversing the anterior (motor) dentate nucleus [28]. The distal contact is placed 5 mm more posterior to the surface of the brainstem, where the outflow tracts of the cerebellum start to coalesce just anterior to the dentate nucleus. Details on targeting are provided in Cajigas et al. Figure 1e [24], and summarized in a recent review [37] highlighting the accuracy of this targeting method.

The day before surgery, participants are assessed by the anesthesia service. On the day of surgery, electrodes are implanted intracranially and the pulse generator is connected and implanted in the infraclavicular region. Brain imaging confirms the appropriate location of the electrodes. Postoperatively and before discharge, participants are followed closely for wound checks, general and neurological examinations, DBS system integrity checks, and brain signal recordings. They are discharged after 2–3 days with the stimulator off.

There will be a visit ten days after surgery to remove surgical staples and for general and neurological examinations. One month after surgery, the DBS system is turned on and programming starts. Participants and caregivers are given a patient programmer to adjust the participant’s DBS settings as needed and will record any adverse events. The participants are evaluated monthly for four months for additional DBS programming to optimize the stimulation settings.

#### Phase 3: N-of-1 trial phase

Following the open-label phase where the stimulation is optimized, the series of N-of-1 trials will be implemented in the experimental phase (Fig. 1). Each trial consists of two one-month periods of either best effective stimulation – determined during the open-label phase – or sham stimulation – ineffective, very low amplitude settings – in alternating order. Patients will be randomly assigned to either sham or effective stimulation as their starting intervention and alternate between the two conditions during the experimental phase. To optimize clinical and research management of participants, a maximum of 2 participants will be studied concurrently but independently. As each trial period ends, another set of 2 participants will be studied, until 5 sets have completed trials. There are 8 possible sequences of 3-cycle combinations of paired arms: AB-AB-AB, AB-AB-BA, AB-BA-AB, AB-BA-BA, and their complements, where A represents effective stimulation and B represents sham stimulation. We will balance the sample by arm and time as follows. To minimize unblinding, concurrent participants will not be assigned to the same sequence: within each set, one of 8 sequences will be randomly assigned to the first participant and one of the remaining 7 sequences to the second participant. Across sets, balance in the starting arm will be maintained by restricting the sequences as needed, so that 5 of 10 participants begin their trial on the intervention arm and 5 on the placebo arm.

Patients and caregivers will be blinded to treatment assignment after the open-label phase. To ensure appropriate blinding of procedures from the investigator’s side, there will be a designated DBS programming clinician who will implement the treatment (effective/sham stimulation) independent from a rating investigator clinician who will assess the outcome measures. The participants will wear a hat/cap to conceal time from surgery for videotaped motor assessments.

#### Phase 4: subsequent follow-up

After the three N-of-1 blocks, the last clinic visit of the study will include repeated neuropsychological testing, QOL and pain questionnaires, videotaped motor assessments, brain recordings, and a second brain MRI. After the conclusion of the study, and for five years following DBS surgery, participants will be invited to return yearly for a research visit for DBS checks and detailed assessments of neurological and behavioral function.

### Participant’s withdrawal

Participants will have the right to withdraw from the trial either fully or partially at any point in time. Follow-up care and testing will be determined before withdrawal to ensure participation is stopped safely. Any data we have already collected from the participant will remain part of the study records. If stimulation is deemed ineffective, surgical removal of the device will be offered to the participant.

### Data management and confidentiality

To ensure confidentiality, participants will be given a research identification number and all data related to any particular participant will be collected and stored using such identification number. Representatives from the National Institutes of Health (NIH), University of California, U.S. FDA, and Office of Human Research Protections (OHRP) may review the data for monitoring or managing study conduct. The study dataset will contain range checks and data validation to ensure data accuracy. Electronic data collection forms will be utilized for each study visit using REDCap, a secure, encrypted, UCSF-sanctioned browser-based metadata-driven electronic data capture system and will be designed to incorporate NINDS Common Data Elements recommendations. The Data Safety Monitoring Board (DSMB) for this study is a panel composed of three external members who will review the study data on a regular basis. Treatment-related adverse events noted by any study personnel classified as definitely, probably, or possibly related to study procedures and as either serious or unexpected, will be reported to the DSMB, IRB, FDA, NIH, and manufacturer.

### Statistical analysis

The primary analysis will use the N-of-1 trials to obtain individual and population-level estimates of the efficacy of cerebellar DBS in ameliorating movement disorder symptoms and in improving QOL for children and young adults with DCP. We will use an intention-to-treat analysis. We anticipate that, within each participant, within-arm CPCHILD and MD-CRS 4-18-R scores measuring the effects of cerebellar stimulation will be relatively stable over time, with slopes approximately parallel with some possible carryover from the effective to sham stimulation arm, based on our prior experience with cerebellar DBS [24]. Based on weekly assessments, each participant will contribute 4 values per arm per block. Within each block, we will treat the participant’s arm-specific values as simultaneous replicates and attribute them to the midpoint of the relevant follow-up interval.

To accommodate alternatives to Gaussian distributions of the measured outcomes, we will analyze them using a generalized linear mixed model (GLMM), with treatment arm, and follow-up time as fixed effects and the subject as a random effect. With 3 blocks per arm, an arm-by-time interaction is unlikely to be needed but will be evaluated in a sensitivity analysis; we will also consider a simplification of the model that substitutes blocks for time. Output of the model will include estimates of the participant-specific mean (95% confidence interval) difference between arms, as well as the overall mean difference (95% confidence interval) between arms and overall plot. An interim analysis of the success of cerebellar stimulation will be performed after the first set of 2 subjects is studied.

### Ethics approval

Ethical approval for the study was given by the University of California Institutional Review Board (IRB Number 22-37182) on January 17, 2024, and the study has obtained FDA Investigational Device Exemption (IDE) status for the use of the Medtronic DBS system for this clinical trial. All protocol amendments will be reported to the study DSMB, NIH, IRB, and the FDA.

## Discussion

Preliminary evidence shows promise of cerebellar stimulation in improving CP-related movement disorders and spasticity [18, 22–27]). This series of N-of-1 trials aims to assess the efficacy of chronic cerebellar stimulation in mitigating movement disorders and enhancing QOL in children and young adults with DCP.

The N-of-1 trial design is particularly useful when treatment effects exhibit variability between individuals [38], as is the case for DBS for complex movement disorders in DCP [10]. N-of-1 designs provide the most rigorous evidence for treatment decisions at the individual level. In addition, when outcomes are aggregated from several patients, N-of-1 trials can yield treatment effect estimates applicable to the broader population, and close to the level of evidence of randomized controlled trials [39]. While not previously used in DBS for dystonia, the N-of-1 design was found to be useful for assessing DBS efficacy in neuropathic pain [40], and it has been more recently recommended for uncommon movement disorders [41].

An assumption for N-of-1 trials is that the treatment to be assessed should have relatively rapid onset and washout, with limited carryover effects. While the beneficial effects of DBS in dystonia following pallidal stimulation may manifest after a delay, non-invasive cerebellar transcranial magnetic stimulation (TMS) and transcranial direct current stimulation (tDCS), and cerebellar DBS appears to have a fast onset and quick wash-out of benefit [24]. If significant delayed-onset and carry-over are observed (during interim analyses), the blinded assessments at the 12-month visit (compared to pre-stimulation baseline) will be used as an endpoint for efficacy. If participants do not tolerate sham or effective stimulation due to resurfacing of dystonia/choreoathetosis, spasticity, and/or pain, we will shorten each block duration by two-to-three weeks. If this is not tolerated, we will continue effective (or sham) stimulation and analyze data for that participant both as intention-to-treat and on an ‘as treated’ basis. The percentage of participants who successfully identify their stimulation settings (effective vs. sham) and are unable to tolerate sham or effective settings will be considered a surrogate secondary endpoint for preliminary effectiveness. In that instance, the effects of the open-label intervention on the blinded video motor ratings and QOL at five months (before the randomized phase) and at 12 months, will be compared to the preoperative baseline.

DBS is considered a minimally invasive surgical option for patients with movement disorders, as it is reversible, adjustable, and has a well-tolerated safety profile. However, given the novelty of the brain target, it is considered an experimental therapy. The risks of intracranial hemorrhage from DBS electrode placement in cerebellar targets are expected to be similar to that for other brain targets (0.2–1.8%) [42]. However, the consequences of intracranial hemorrhages in the posterior fossa are greater for a given size of hematoma, and in the event of bleeding, carry a higher risk for the need for surgical decompression. Protection from these surgical risks includes routine neurosurgical practices such as preoperative prophylactic antibiotics, strict intra-operative control of blood pressure, and preoperative checks of coagulation factors. Intraoperative computed tomography CT will be used to confirm the intended placement. The risk of brainstem dysfunction post-operatively is reduced by not advancing the lead all the way to the edge of the brainstem. The youngest participant’s age will be 7 at time of surgery. At this age, no additional growth of cranial structures is expected to be enough to pose a threat to lead placement. Lead migrations in children 7 and older with cerebral palsy are not any more common than in adult populations [43]. Additionally, any potential lead migrations would be expected to be posteriorly, away from the brainstem, posing less risk to brainstem structures.

Since cerebellar stimulation is a relatively novel target, cerebellar stimulation-related side effects are unknown. Some potential adverse effects during cerebellar stimulation may include stimulation-induced dysarthria, hypotonia, and ataxia. To prevent these risks, a wide range of symptoms is consistently monitored by movement disorder specialists.

Brain recordings may also shorten the lifespan of the implanted battery, requiring an earlier-than-expected replacement. Our data collection will be limited to one- hour maximum per session, which would only reduce the implantable pulse generator’s longevity by a few days [44].

Additionally, the use of DBS in children has its own bioethical, social, and legal considerations, and evidence from the adult DBS literature may not be readily translatable to children [45]. This study will enroll vulnerable populations, including children seven years and older and individuals who may have intellectual disabilities. The rationale for involving these populations is the need for better therapies to improve movement disorders, tone, and QOL for them. Only those patients refractory to prior treatments and considered adequate candidates for DBS are recruited. First-hand evidence in these vulnerable groups is needed, as children and young adults with dyskinetic CP are rarely included in trials. Their exclusion may deprive them from the benefits of research and expose them to additional risks if interventions are later used on them without adequate data [46].

This trial has primary objectives to assess the feasibility and safety of cerebellar DBS in addressing movement and tone disorders in children and young adults with DCP. Clinical outcomes include average total CPCHILD score and average total MD-CRS 4-18-R scores, and key secondary outcome measures include other health-related QOL scores, other motor and spasticity ratings, and exploratory automatic video kinematic analyses. The information and outcomes gathered from this study will be pivotal for establishing the principles and generating a robust methodological framework to develop DBS therapy in DCP.

## Data Availability

No datasets were generated or analysed during the current study.

## Abbreviations

CP: Cerebral Palsy
DCP: Dyskinetic/Dystonic Cerebral Palsy
DBS: Deep Brain Stimulation
FDA: U.S. Food and Drug Administration
QOL: (Health-Related) Quality of Life
HIE: Hypoxic Ischemic Encephalopathy
GMFCS: Gross Motor Function Classification System
CPCHILD: Caregiver Priorities & Child Health Index of Life with Disabilities
MD: CRS-4-18-R-Movement Disorder Childhood Rating Scale 4-18 Revised
NIH: National Institutes of Health
DSMB: Data Safety and Monitoring Board
